# White matter microstructure of superior longitudinal fasciculus II is associated with intelligence and treatment response of negative symptoms in patients with schizophrenia

**DOI:** 10.1038/s41537-022-00253-9

**Published:** 2022-04-27

**Authors:** Joonho Lee, Jong-Soo Oh, Chun-Il Park, Minji Bang, Gihye Sung, Sra Jung, Sang-Hyuk Lee

**Affiliations:** 1 Department of Psychiatry, CHA Bundang Medical Center, CHA University, Seongnam, Republic of Korea; 2 CHA University School of Medicine, Seongnam, Republic of Korea; 3 Department of Psychology, Korea University, Seoul, Republic of Korea; 4 Department of Psychiatry, Kangbuk Samsung Medical Center, Sungkyunkwan University School of Medicine, Seoul, Republic of Korea

**Keywords:** Schizophrenia, Schizophrenia

## Abstract

Although the potential role of superior longitudinal fasciculus (SLF) in intellectual deficits and treatment response (TR) in patients with schizophrenia (SZ) has been previously described, little is known about the white-matter (WM) integrity of SLF subcomponents (SLF I, II, III, and arcuate fasciculus) and their particular relationships with the clinical presentations of the illness. This study examined the associations between fractional anisotropy (FA) of SLF subcomponents and intelligence level and 6-month treatment response (TR) of negative symptoms (NS) in patients with SZ. At baseline, 101 patients with SZ and 101 healthy controls (HCs) underwent structural magnetic resonance imaging. Voxel-wise group comparison analysis showed significant SLF FA reductions in patients with SZ compared with HCs. Voxel-wise correlation analyses revealed significant positive correlations of FAs of right SLF II with Korean–Wechsler Adult Intelligence Scale at baseline and the percentage reduction of negative syndrome subscale of the Positive and Negative Syndrome Scales at 6 months. These findings suggest that aberrance in WM microstructure in SLF II may be associated with intellectual deficits in patients with SZ and TR of NS, which may support the potential role of SLF II as a novel neuroimaging biomarker for clinical outcomes of the illness.

## Introduction

Schizophrenia (SZ) is a debilitating psychiatric syndrome characterized by positive and negative symptoms (NS), and cognitive impairment. Pharmacological interventions, such as antipsychotic medications, are generally effective in treating positive symptoms; however, they have only limited effects on NS and cognitive impairment, which are the cardinal features of SZ largely responsible for long-term morbidity and poor occupational, social, and economic functioning in these patients^1,2^. Although often discussed separately, the negative and cognitive symptoms of SZ are increasingly being considered to originate in similar neural bases due to their shared features^1^. Previous studies^3–5^ have consistently reported widespread white-matter (WM) abnormalities and suggested the implications of disrupted neural networks (e.g., frontocortico-temporal, fronto-striatal, and fronto-temporal-parietal) on NS and cognitive deficits in SZ. More specifically, a recent meta-analysis^6^ of 59 individual studies found extensive alterations in WM bundles in the corpus callosum, thalamic radiations, inferior fronto-occipital fasciculus, uncinate fasciculus, cingulum, and superior longitudinal fasciculus (SLF) in patients with SZ.

The SLF is a major association fiber that encompasses the frontal, parietal, and temporal areas^7^. It has been implicated in patients with SZ largely because of its important role in cognitive functioning, including attention and memory^8^. For instance, a structural magnetic resonance imaging (MRI) study^9^ reported a significant association between the intelligence quotient and frontotemporal cortex in patients with first-episode psychosis, suggesting the involvement of cortical structures interconnected via the SLF in cognitive deficits presented in these patients. Additionally there is direct evidence that abnormalities in the SLF are associated with cognitive impairment in patients with SZ, and a previous diffusion tensor image (DTI) study^10^ demonstrated WM disruption in the SLF and its association with impaired verbal working memory tasks in patients with recent-onset SZ. Another recent DTI study^11^ reported a positive correlation between processing speed and fractional anisotropy (FA) values in the right SLF. Recent studies have also suggested a link between WM alterations in the SLF and NS in individuals at an ultra-high risk of psychosis^12,13^, those with first-episode psychosis^14^, and those with SZ^15,16^. Collectively, the findings from these studies indicate that SLF may be involved in the pathophysiology of cognition and NS in SZ.

Recent studies in literature have also proposed that SLFs may have implications in the clinical outcomes of SZ. In their DTI-tractography study^14^, Luck et al. reported that first-episode psychosis patients with poor outcomes showed greater disruptions in the frontotemporal WM microstructures (uncinate and SLF) than those with good outcomes. They speculated that smaller myelin alterations in patients with good outcomes, as reflected by higher FAs in these structures, might have facilitated a better response to antipsychotic medications. Another DTI study^17^ of patients with first-episode SZ demonstrated that improvement in disruptions in widespread WM tracts (indicated by decreased FAs and increased mean diffusivity [MD] and radial diffusivity [RD]), including the left SLF, correlated with improvement in psychosis and processing speed at 8 weeks from baseline. Thus, evidence from these studies suggests that WM disruptions in the SLF may be a key feature in predicting treatment response (TR) in patients with SZ, warranting further longitudinal studies with larger clinical cohorts to corroborate these findings.

Since the SLF connects a wide range of brain regions and is involved in a variety of associative and higher brain functions, researchers have suggested structural delineation of this large fiber system. Makris et al.^18^ discovered that the four subdivisions of the SLF observed in non-human primates, SLF I, SLF II, SLF III, and arcuate fasciculus (AF), could be identified in the human brain and tentatively proposed functional roles of each SLF subcomponent in humans. SLF I, which interconnects the medial/superior parietal regions with the dorsal premotor region, may contribute to higher levels of motor regulation. SLF II forms a major link between the prefrontal and parietal lobes, and may help these regions communicate visual information, thus contributing to the regulation of visuospatial attention. SLF III, which connects the rostral part of the inferior parietal lobule with the lateral inferior frontal lobe, is speculated to transfer somatosensory information such as language articulation. AF runs contiguously with SLF II and may help the prefrontal cortex to modulate audiospatial information. Subsequent multimodal studies^19,20^ have further contributed to our understanding of the functional roles of the SLF subcomponents in the human brain. However, there is a lack of studies investigating specific relationships between the structural aberrations of SLF subcomponents and their implications on the symptomatology of SZ.

Hence, this study sought to investigate the aberrant WM microstructure of SLF subcomponents and its association with intellectual deficits and TR of NS in patients with SZ. Our hypotheses were as follows:

- There would be disrupted WM integrity in the SLF (observed as lower FAs) in patients with SZ at baseline, when compared to healthy controls (HCs).
- In patients with SZ, intelligence level at baseline and 6-month TR of NS would be positively associated with FAs of SLF areas that showed significant differences between the two groups at baseline.
- Distinct patterns of associations with intelligence level at baseline and 6-month TR of NS would be present in each SLF subcomponent, namely SLF I, II, III, and AF.

## Results

### Demographic and clinical characteristics of the study participants

Table 1 displays the demographic and clinical profiles of patients with SZ and HCs. There were no significant differences in sex or age between the two groups. The years of education and total intracranial volume were significantly lower in patients with SZ than in HCs. The mean age of patients with SZ was 37.0 ± 11.5 years, and the median duration of illness was 10 months. Full-scale intelligence quotient (FSIQ) and all subtest scores of Korean–Wechsler adult intelligence scale (K-WAIS) except “information” were significantly lower in patients with SZ than in HCs. Table 2 describes the severity of clinical symptoms in patients with SZ at baseline and 6 months, assessed using the Positive and Negative Syndrome Scale (PANSS). No significant differences in demographic and clinical characteristics were found between patients with (*n* = 78) and without (*n* = 23) K-WAIS assessments.

**Table 1:** Demographic and clinical characteristics of the study participants.

	Patients with SZ (*N* = 101)	HCs (*N* = 101)	Statistics	*p*
Sex				
Male, *n* (%)	35 (34.7)	42 (41.6)	*χ*^2^ = 1.45	0.311
Female, *n* (%)	66 (65.3)	59 (58.4)		
Age (years), mean ± SD	37.0 ± 11.5	37.9 ± 8.7	*t* = 0.63	0.521
Education (years), mean ± SD	13.0 ± 2.4	16.7 ± 2.5	*t* = 10.40	<0.001
Duration of illness (months), mean ± SD	46.3 ± 72.2			
Intracranial volume (mL), mean ± SD	1466.9 ± 139.7	1543.5 ± 130.4	*t* = 4.01	<0.001
Antipsychotic medications at MRI scan (mg/day), mean ± SD^a^	435.3 ± 256.5			
Antipsychotic medications at 6 months (mg/day), mean ± SD^a^	715.0 ± 1634.1			
Duration of antipsychotic medication before scan of antipsychotic-naive or free participants (days), mean ± SD	6.8 ± 7.2			
Types of antipsychotics				
Paliperidone (*n*)	32			
Olanzapine (*n*)	2			
Risperidone (*n*)	27			
Amisulpride (*n*)	32			
Aripiprazole (*n*)	8			
Full-scale intelligence quotient, mean ± SD	88.6 ± 18.5	114.9 ± 6.3	*t* = 4.23	<0.001
Verbal intelligence quotient	94.7 ± 16.2	117.0 ± 9.0	*t* = 7.41	<0.001
Information	9.3 ± 3.3	11.1 ± 2.5	*t* = 1.40	0.166
Digit span	8.5 ± 3.4	11.6 ± 1.7	*t* = 2.27	0.026
Vocabulary	9.2 ± 3.3	12.8 ± 1.1	*t* = 6.99	<0.001
Arithmetic	8.0 ± 2.9	12.1 ± 2.6	*t* = 2.93	0.004
Comprehension	9.9 ± 3.3	13.7 ± 0.9	*t* = 6.74	<0.001
Similarity	9.4 ± 3.4	13.9 ± 1.6	*t* = 2.73	0.008
Performance intelligence quotient	89.7 ± 17.5	110.7 ± 7.6	*t* = 7.72	<0.001
Picture completion	8.4 ± 2.9	10.1 ± 2.0	*t* = 4.51	<0.001
Picture arrangement	8.9 ± 2.9	11.0 ± 1.4	*t* = 2.02	0.049
Block design	8.6 ± 3.9	13.7 ± 1.6	*t* = 5.59	<0.001
Object assembly	9.2 ± 3.8	12.6 ± 2.1	*t* = 3.21	0.008
Digit symbol	9.0 ± 3.0	12.2 ± 1.2	*t* = 2.54	0.014

*SZ* schizophrenia, *HCs* healthy controls, *SD* standard deviation, *MRI* magnetic resonance imaging.

^a^Chlorpromazine equivalent doses were calculated according to Gardner et al.^61^.

**Table 2:** Positive and Negative Syndrome Scale at baseline and at 6 months in patients with schizophrenia.

Mean ± SD	PANSS score at baseline (*N* = 101)	PANSS score at 6 months (*N* = 95)
PANSS composite score	116.5 ± 26.7	53.3 ± 12.3
Positive scale	29.4 ± 7.5	11.1 ± 3.9
Negative scale	27.2 ± 8.3	14.4 ± 4.0
General psychopathology scale	59.9 ± 13.4	27.8 ± 5.9

*SD* standard deviation, *PANSS* Positive and Negative Syndrome Scale.

### Voxel-wise comparison of DTI indices of SLF between patients with SZ and HCs

**Fig. 1:**
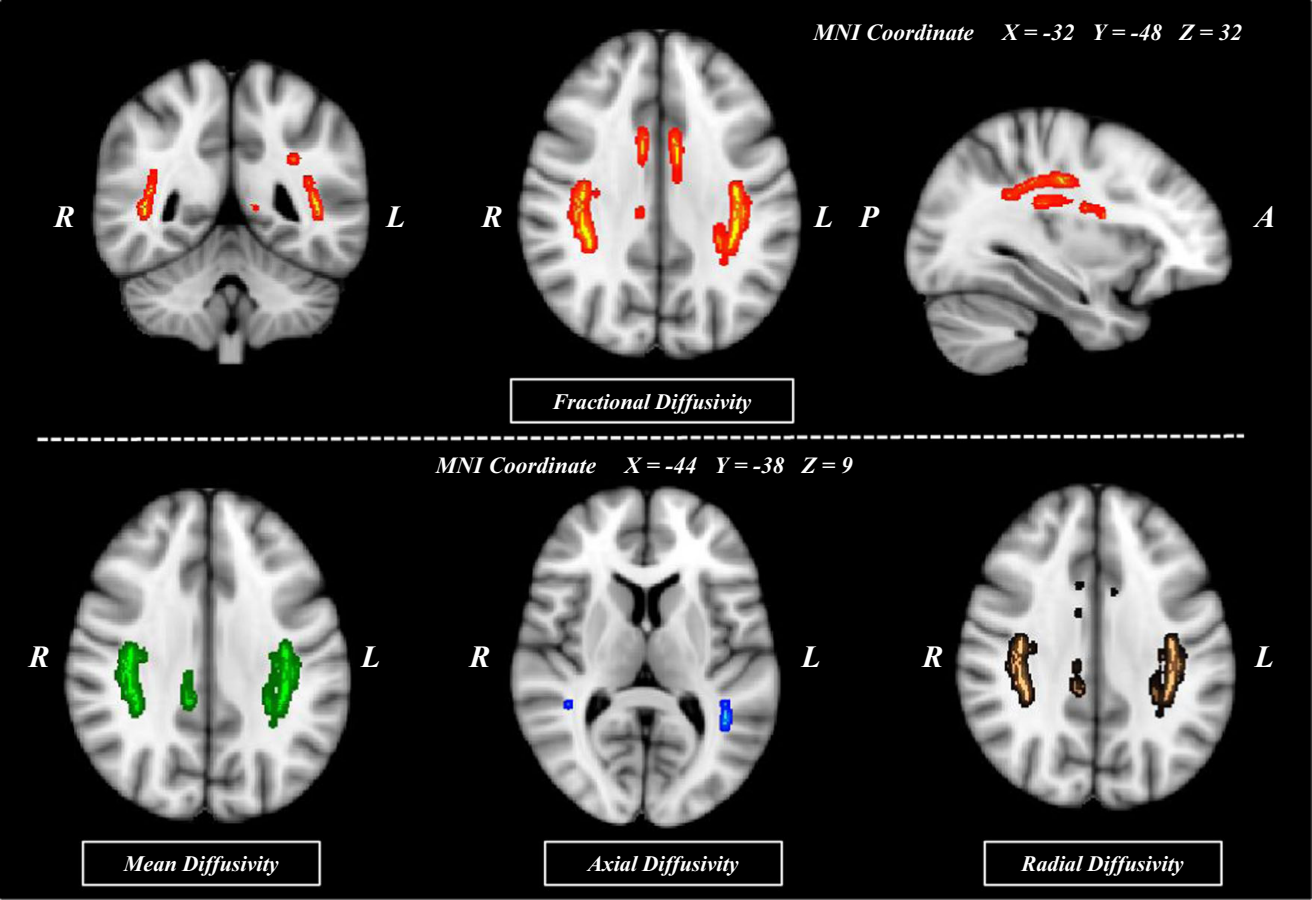
Comparison of the regions showing significant changes in the fractional anisotropy, mean diffusivity, axial diffusivity, and radial diffusivity values between patients with schizophrenia and healthy controls (*N* = 101 for both). Voxels demonstrating significantly (threshold-free cluster enhancement, *p* = 0.05; family-wise error corrected) decreased fractional anisotropy values in patients with schizophrenia as compared to healthy controls are shown in red-yellow color. Significantly increased mean diffusivity, axial diffusivity, and radial diffusivity values in patients with schizophrenia as compared with healthy controls are shown in green, blue, and brown, respectively. The number of permutations was 1000. (MNI, Montreal Neurologic Institute; R, right; L, left; P, posterior; A, anterior).

Figure 1 shows the voxel-wise comparison analyses for SLF FAs, along with MDs, axial diffusivity (AD)s, and RDs, between patients with SZ and HCs. FAs were significantly lower in patients with SZ for voxels in extensive areas of the SLF (*p* < 0.002; family-wise error [FWE] corrected). MD, AD, and RD were significantly higher in patients with SZ than in HCs (*p* < 0.003, *p* < 0.009, and *p* < 0.002, respectively; FWE corrected). Inspection of the areas with respect to the regions of interest (ROI) mask for SLF subcomponents demonstrated that voxels of significant between-group FA differences were identified in all eight SLF subcomponents. When controlling for age, sex, and years of education as covariates, voxel-wise comparison analyses demonstrated lower FAs for all SLF subcomponents in patients with SZ (*p* < 0.002; FWE corrected). The significance of the results remained the same after the exclusion of 14 patients with SZ who were on antipsychotic medications at the time of enrollment. For a comprehensive analysis of the SLF, repeated measures analysis of variance (RM-ANOVA) was performed in addition to voxel-wise analysis (Supplementary Methods and Results).

### WM microstructures of SLF subcomponents and their associations with intelligence level in patients with SZ

**Fig. 2:**
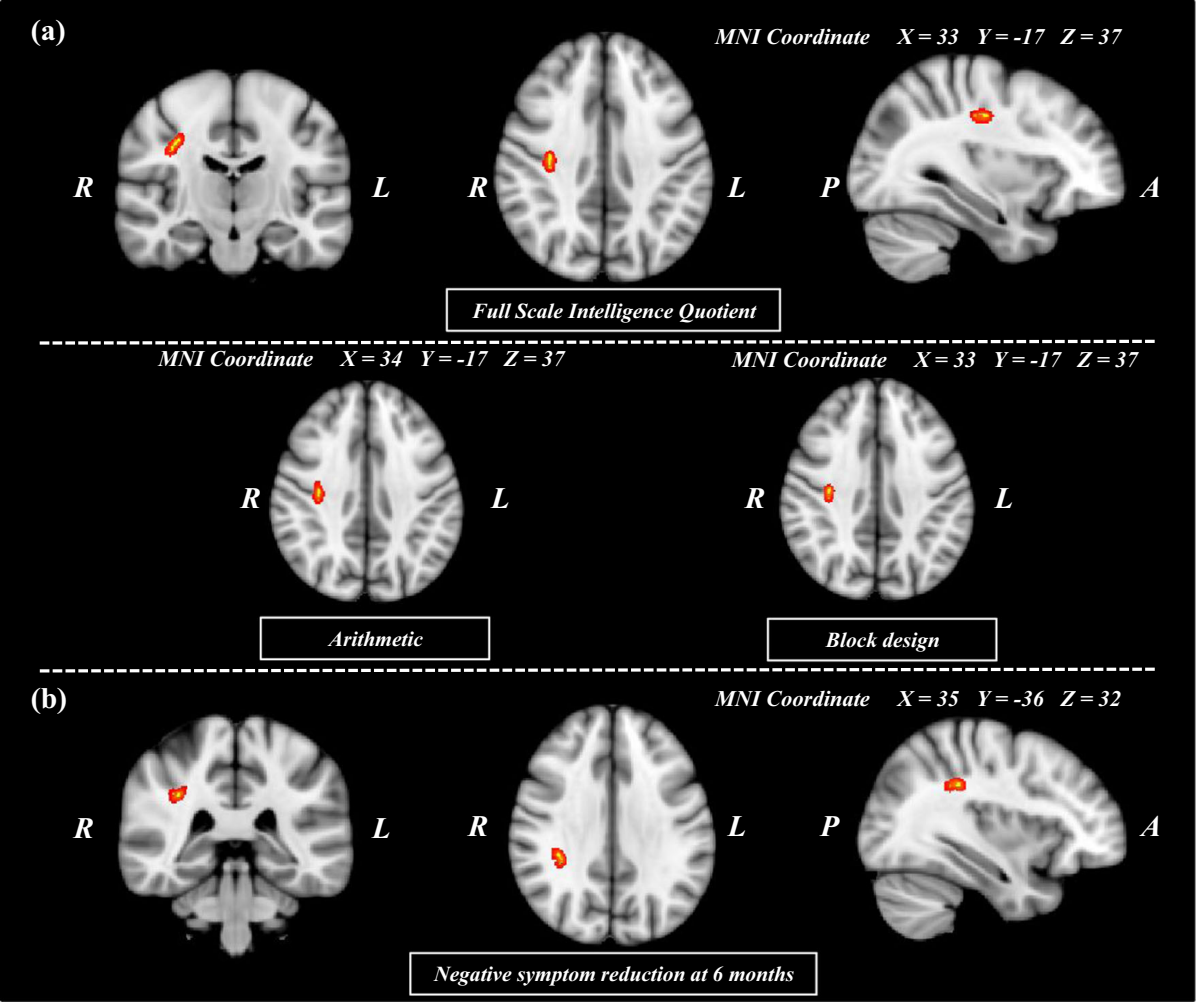
Regions showing positive correlation between fractional anisotropy values and intelligence quotient scores and negative symptom reduction at 6 months in patients with schizophrenia. Voxels demonstrating regions showing significant (threshold-free cluster enhancement, *p* = 0.05; family-wise error corrected) positive correlation between the fractional anisotropy values and the intelligence quotient (**a**) and treatment response (**b**) in patients with schizophrenia are depicted by red-yellow circles. The number of permutations was 1000. (MNI, Montreal Neurologic Institute; R, right; L, left; P, posterior; A, anterior).

Voxel-wise correlation analyses showed positive correlations between FSIQ and FAs in areas within the right SLF II in patients with SZ (*p* < 0.004; FWE corrected). The significance of the results remained the same after controlling for age, sex, years of education, and antipsychotic medication use at baseline as covariates. No significant correlation was observed in HCs. Exploratory voxel-wise correlation analyses for K-WAIS subtest scores showed that FAs of right SLF II were positively correlated with “digit span”, “vocabulary,” “arithmetic,” “comprehension,” “similarities,” “picture arrangement,” and “block design” subscales in patients with SZ (Fig. 2a). Multivariate multiple stepwise linear regression analysis (*F* = 14.12, *p* < 0.001) showed that the FAs of the right SLF II were independently correlated with “arithmetic” (*β* = 0.016, 95% CI = 0.002–0.029, *p* = 0.024) in working memory dimension and “block design” (*β* = 0.012, 95% CI = < 0.001–0.024, *p* = 0.045) in visuospatial dimension.

### WM microstructures of SLF subcomponents and their associations with 6-month TR of NS in patients with SZ

Figure 2b depicts voxel-wise correlation analyses showing positive correlations between the 6-month TR of NS and FAs of areas within the right SLF II in patients with SZ (*p* < 0.04; FWE corrected). Pearson correlation analysis also demonstrated a significant positive correlation between the 6-month TR of NS and the FAs of the right SLF II (*r* = 0.420, *p* < 0.001; Supplementary Fig. 2). The significance of the results remained the same after excluding 4 patients who received long-acting injectable antipsychotics at 6 months and controlling for age, sex, years of education, and antipsychotic medication at 6 months as covariates. No significant associations were demonstrated for the total PANSS scores and the positive and general psychopathology subscales.

Multivariate multiple stepwise linear regression analysis (*F* = 11.09, *p* < 0.001) showed that the 6-month TR of NS was explained by the FAs of the right SLF II (*β* = 276.16, 95% CI = 107.07–445.26, *p* = 0.002), as well as sex (*β* = 21.90, 95% CI = 7.63–36.18, *p* = 0.003) and baseline PANSS scores (*β* = 0.58, 95% CI = 0.32–0.84, *p* = 0.003), indicating a significant predictive power of FAs of the right SLF II for TR of NS.

## Discussion

To our knowledge, this is the first study to investigate the aberrant WM microstructure of SLF subcomponents and its association with intelligence level and TR in patients with SZ. In this study, patients with SZ showed significantly lower FAs in all SLF subcomponents in both hemispheres than HCs. We also demonstrated positive correlations in patients with SZ between FAs of right SLF II and FSIQ, as well as K-WAIS subtests in working memory (“arithmetic”) and perceptual organization (“block design”) and TR of NS at 6 months.

Previously, DTI studies^10,21–24^ have consistently reported reduced FAs in the tracts connecting the frontal cortex with the temporal and parietal cortices in patients with SZ, suggesting widespread WM disintegration during the course of illness. Our findings of lower FAs in all SLF subcomponents of patients with SZ, as compared to HCs, are consistent with those of previous DTI studies^24–26^ that reported WM disruptions in the SLF as a whole. However, there is a lack of studies specifically investigating the structural aberrations of the WM tracts in each SLF subcomponent in patients with SZ. DTI studies of healthy individuals^20^ have suggested distinct functions of the SLF subcomponents in accordance with their respective brain regions. SLF I originates from the superior parietal lobe and terminates within the supplementary motor and premotor areas in the frontal lobe, thus serving a function in proprioception and motor movements. SLF II connects the posterolateral parietal lobe to the dorsolateral prefrontal cortex and has been suggested to be involved primarily in visuospatial awareness and attention. SLF III originates from the supramarginal gyrus, terminates within the dorsal prefrontal cortex, and plays a role in somatosensory input, fine movements, phonetics, and language articulation. Lastly, the AF connects the temporal, frontal, and parietal lobes and is known to play a major role in speech processing. Our findings of WM alterations across all SLF subcomponents in patients with SZ may have some implications for the wide spectrum of symptomatology of this illness. These include (1) disorders of self-awareness in the context of dysfunctional proprioception;^18,27^ (2) abnormal visuospatial processing, which has been suggested to contribute to the development of positive symptoms;^28^ and (3) reduced ability to comprehend language, which may be associated with positive formal thought disorders^29^, verbal hallucinations, and delusions^30,31^. However, it is noteworthy that these arguments remain largely speculative and are beyond the scope of our study, warranting further studies to shed light on the specific associations between the disturbances in WM microstructures of SLF subcomponents and the various symptoms of SZ.

The positive correlation noted between FAs of the right SLF II and FSIQ in patients with SZ corroborates previous studies^32–34^ that reported an association between SLF disruptions and cognitive functions. In our study, voxel-wise correlation analyses and exploratory multiple linear regression for K-WAIS subtests also revealed that FAs of right SLF II were associated independently with “arithmetic” and “block design” subtests. This finding is consistent with that of a recent DTI study^35^ on healthy individuals, which reported significant associations between SLF FAs and working memory and perceptual organization. Although the specific role of each SLF subcomponent in intelligence remains uncertain, evidence supports the involvement of SLF II in executive function, including working memory. In their study using voxel-based lesion-symptom mapping, Kinoshita et al.^36^ proposed that both the frontal and parietal terminations of SLF II (along with SLF I) could be more specifically associated with spatial working memory. It can be speculated that disrupted microstructures in SLF II, a major domain of the dorsal pathway of attention^20^, may contribute to working memory deficits in patients with SZ. This is supported by our finding of significant correlations between FAs of the right SLF II and “arithmetic” subtest scores, which typically represent working memory functioning. Moreover, the positive correlations found between FAs of the right SLF II and “block design” subtest scores, which correspond to perceptual organization^37^, may be interpreted as further evidence supporting the notion that SLF II, especially in the non-dominant hemisphere, subserves the function of visuospatial awareness^38,39^. Notably, the region in the right SLF II, which showed a significant correlation with “arithmetic” subtest scores, extended over the WM regions from the precentral (frontal lobe) to the postcentral and supramarginal gyri (parietal lobe). On the other hand, such a result was shown only in the WM near the precentral gyrus for subtest scores of “block design”. This may be relevant in understanding the potential role of SLF II with respect to parietal and frontal lobe involvement in working memory performance, as discussed in previous studies^40^. Thus, these findings suggest the involvement of SLF II in the cognitive deficits obesrved in patients with SZ, specifically in working memory and perceptual organization.

Our study also demonstrated a positive correlation between FAs of the right SLF II and TR of NS at 6 months in patients with SZ. Findings from previous studies have generally supported the notion that WM alterations are associated with clinical outcomes in patients with SZ. For instance, Kochunov et al.^41^ reported that the WM regional vulnerability index, a measure of agreement between FA values at the individual level and the expected pattern from the ENIGMA study, correlated with processing speed and negative symptoms. This indicates a heightened risk of treatment resistance among patients with SZ who exhibit more severe WM impairments. A recent DTI study^42^ in patients with first-episode SZ reported that those with poor outcomes (<50% reduction in PANSS scores at 1 year) showed significantly different patterns of baseline FAs in various WM tracts, including the right SLF, as compared to those with good outcomes. This provides further evidence that WM microstructures may be relevant in the prediction of clinical prognosis in patients with SZ. WM abnormalities displayed as a pattern of decreased FAs and increased RDs and MDs, similar to that observed in the right SLF II of patients with SZ in our study, might be interpreted as a result of demyelination^43^, although the precise biological processes underlying this finding should be further investigated. In vitro and in vivo studies^44–46^ have suggested that atypical antipsychotics target the development of oligodendrocytes by promoting their proliferation and differentiation, thus potentially facilitating the reversal of myelin deficits that may contribute to the pathogenesis of SZ^47^. Hence, it can be proposed that the beneficial effects of atypical antipsychotics on oligodendrocytes are lost in patients with SZ with more pronounced WM demyelination in the right SLF II, resulting in a poorer TR to antipsychotics. Furthermore, it may be relevant to address the findings of previous DTI studies^12,48^ that reported a significant positive correlation between changes in SLF FAs and those in NS in individuals at an ultra-high risk of psychosis. In the aforementioned studies, Krakauer et al. proposed that aberrant WM maturation process in SLF could be responsible for the SZ symptomatology, particularly NS. Although differing in directionality, our findings support the argument that the SLF may be involved in the development of NS in SZ.

The results of this study may expand our knowledge regarding the role of the SLF, particularly SLF II, in cognitive impairment and NS in patients with SZ. The strengths of our study include a relatively large cohort, high follow-up rate, and the use of voxel-wise permutation analyses with secondary analyses to corroborate our findings.

This study had several limitations. First, all patients with SZ underwent DTI after the initiation of antipsychotic treatment. Although majority of them were drug-naive or drug-free (86.1%) and the mean duration of antipsychotic treatment before DTI was relatively short (6.8 ± 7.2 days), it is unclear whether the effect of antipsychotic drugs was negligible on the WM structures. Although post hoc subgroup analyses for drug-naive patients with SZ showed the same significant result as the original analysis, future investigations with a larger number of drug-naive patients are required to exclude the confounding effect of antipsychotic medication. Second, although all patients in this study were provided with fairly consistent treatment, it is necessary to consider that possible individual differences in treatment, such as supportive psychotherapy, might have confounded treatment outcomes. Third, our results from voxel-wise analyses should be interpreted with caution, due to potential sources of type I errors uncontrolled for multiple corrections, for example multiple time points of clinical assessments. Fourth, while the focus of this study was to investigate the WM microstructure of SLF subcomponents, a whole brain analysis may be necessary for a more comprehensive understanding of the neural correlates of cognitive deficits and NS in patients with SZ. Finally, future longitudinal studies with DTI data at multiple time points are warranted to investigate structural changes in the SLF during the course of SZ.

In conclusion, we demonstrated disrupted WM integrity (measured as lower FAs) of all SLF subcomponents in patients with SZ, as well as associations between altered WM microstructure of SLF II and poorer intellectual functioning and TR of NS in patients with SZ. These findings suggest that SLF, particularly SLF II, may contribute to cognitive deficits in patients with SZ, and indicate a potential role of SLF II as a neuroimaging biomarker for clinical outcomes of NS.

## Methods

### Participants

**Fig. 3:**
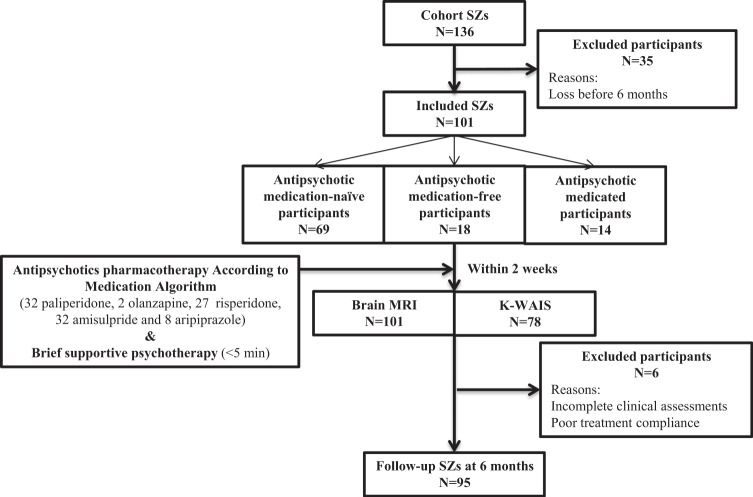
Flow-chart showing participants included in this analysis. Flow-chart showing recruitment of patients with schizophrenia in this study (SZ, schizophrenia; MRI, magnetic resonance imaging; K-WAIS, Korean–Wechsler Adult Intelligence Scale).

Figure 3 depicts a flowchart of the enrollment of participants in the present study. Participants in the CHA SZ cohort study were recruited from patients with SZ who received either inpatient or outpatient treatment at the Department of Psychiatry, CHA Bundang Medical Center, Seongnam, Republic of Korea, between February 2014 and September 2018. Patients with SZ were enrolled after having been diagnosed with SZ by experienced psychiatrists, in accordance with the Diagnostic and Statistical Manual of Mental Disorders, 4th edition, text revision (DSM-IV-TR) and the structured clinical interview for DSM-IV-TR Axis I Disorders^49^. A total of 136 patients with SZ were recruited for the CHA SZ cohort study and underwent good-quality MR diffusion imaging at baseline.

One-hundred one of the 136 patients with SZ, who had continued pharmacological treatment for at least 6 months, were included in this study. At baseline, 69 of 101 patients with SZ (68.3%) were antipsychotic-naive, 18 (17.8%) were free of antipsychotics for at least 6 months, and the remaining 14 (13.9%) were on antipsychotics at the time of enrollment. Pharmacological treatment for all patients with SZ was provided in accordance with the Korean Medication Algorithm for SZ^50^ or Clinical Practice Guidelines: Treatment of SZ^51^. Psychological interventions provided to study participants were confined to brief supportive psychotherapy (<5 min). Six patients were excluded from the correlation analyses for TR of NS at 6 months due to incomplete clinical assessments.

Healthy individuals were recruited from the local community by using online and print advertisements. In this study, 101 individuals with no personal or first-relative family history of psychiatric disorders were included as HCs. The exclusion criteria for study enrollment were as follows: (1) current or past history of substance use disorders; (2) neurological diseases; (3) intellectual disability; and (4) head trauma with loss of consciousness. Left-handed individuals were also excluded based on the Edinburgh Handedness Inventory^52^.

All study procedures were approved by the Institutional Review Board of CHA Bundang Medical Center and adhered to the latest version of the Declaration of Helsinki and principles of Good Clinical Practice. (Approval number: 2019-05-030-009) All study participants and parents/guardians of minor participants (<18 years of age) provided written informed consent following a thorough explanation of the study procedures.

### Clinical assessments

The intelligence level was assessed by trained clinical psychologists using the K-WAIS^53^ in patients with SZ and HCs. A detailed summary of K-WAIS scores in the two groups, as well as subtest scores for verbal IQ (information, digit span, vocabulary, arithmetic, comprehension, and similarities) and performance IQ (picture completion, picture arrangement, block design, object assembly, and digit symbols) are shown in Table 1.

The severity of clinical symptoms in patients with SZ was assessed by experienced psychiatrists using the PANSS^54^ at baseline and at 6 months. TR of NS was defined as the percentage reduction in the PANSS negative syndrome subscale from baseline to 6 months. In accordance with recent suggestions by Leucht et al.^55^ to use 0–6 scaling system instead of 1–7, “PANSS negative syndrome subscale at baseline −7” was used for the denominator of the fraction presented below.

$${{{\mathrm{Treatment}}}}\;{{{\mathrm{Response}}}}\left( {{{\mathrm{\% }}}} \right) = \frac{{\left[ {{{{\mathrm{PANSS}}}}\;{{{\mathrm{Negative}}}}\;{{{\mathrm{at}}}}\;{{{\mathrm{baseline}}}}} \right] - [{{{\mathrm{PANSS}}}}\;{{{\mathrm{Negative}}}}\;{{{\mathrm{at}}}}\;6\;{{{\mathrm{months}}}}]}}{{[{{{\mathrm{PANSS}}}}\;{{{\mathrm{Negative}}}}\;{{{\mathrm{at}}}}\;{{{\mathrm{baseline}}}}] - 7}} \times 100$$

### Neuroimaging data acquisition

MRI scanning was performed for all study participants using a 3.0 T GE Signa HDxt scanner (GE Healthcare, Milwaukee, WI, USA) with an eight-channel phase-array head coil at CHA Bundang Medical Center, CHA University. MRI scans were acquired within two weeks of the initiation of antipsychotic medications for all antipsychotic-naive/free patients with SZ. The mean duration of pharmacotherapy before MRI of the drug-naive or drug-free participants was 5.09 ± 5.73 days. An echo-planar imaging (EPI) sequence was utilized for diffusion-weighted images with the following parameters: repetition time of 17,000 ms, echo time of 108 ms, field of view of 24 cm, 144 × 144 matrix, 1.7 mm slice thickness, and voxel size of 1.67 × 1.67 × 1.7 mm^3^. The eddy current effects were minimized by applying the double-echo option. To reduce the impact of EPI spatial distortions, an eight-channel coil and an array of spatial sensitivity encoding techniques (ASSET, GE Healthcare) with a sensitivity encoding speed-up factor of two were used. Seventy axial slices parallel to the anterior commissure–posterior commissure line covering the entire brain in 51 directions with a *b*-value of 900 s/mm^2^ and eight baseline scans with a *b*-value of 0 s/mm^2^ were acquired. From the diffusion-weighted images, the DTIs were approximated using the least-squares method (approximate scan time: 17 min). The average quality metrics of diffusion-weighted images from all participants, computed using the quality assessment method described by Roalf et al.^56^, were found to range in the “good” and “excellent” groups.

### Tract-based spatial statistics analysis

Tract-based spatial statistics (TBSS, version 1.2) in the Functional MRI of the Brain (FMRIB) Software Library (FSL, version 6.0, Oxford, UK, http://www.fmrib.ox.ac.jk/fsl) were used for the statistical analysis of FAs according to the standard procedure^57^. The FSL of the FA images was used to proceed with the nonlinear regression and skeletonization stages to obtain ADs, RDs, and MDs, as well as to estimate the projection vectors onto the mean FA skeleton from each individual participant. Nonlinear distortion and skeleton projection can also be applied to other types of images. DTI preprocessing, including skull stripping using a brain extraction tool and eddy current correction, was performed using FSL. FA images were constructed by fitting a tensor model to the raw diffusion data^58^. The FMRIB nonlinear image registration tool was used to align the FA data of all participants in the standard space (Montreal Neurologic Institute 152 standard). All transformed FA images were combined and applied to the original FA map, resulting in a standard-space version. All adjusted images of FAs were averaged to generate an image of the mean FA, which was thinned (skeletonized) and used only at the center of the WM tract to produce a mean FA skeleton. To include only major fiber bundles, the threshold was set at FA > 0.2 (TBSS default). Voxel-wise permutation-based nonparametric inference was performed on the skeletonized FA data using FSL Randomize. Independent *t*-test and general linear model regression analysis were performed with 1000 permutations, and the significance level was set at *p* < 0.05, and corrected for the FWE rate. To avoid making an arbitrary choice of the cluster-forming threshold, threshold-free cluster enhancement with multiple comparison correction was used, while preserving the sensitivity benefits of cluster-wise correction.

### Generation of masks of SLF and subcomponents

**Fig. 4:**
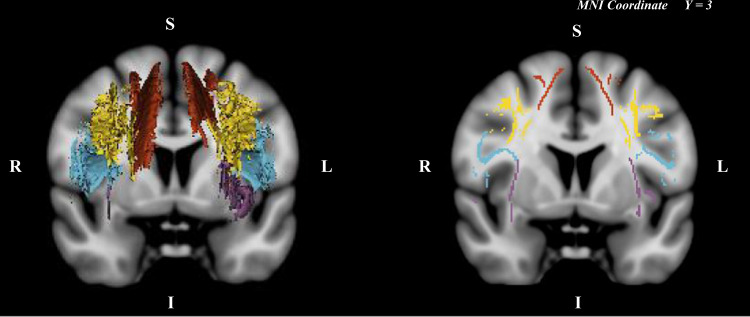
Manually edited SLF subcomponents. Regions of interest in both hemispheres extracted using 3D Slicer Red = superior longitudinal fasciculus I; Yellow = superior longitudinal fasciculus II; Blue = superior longitudinal fasciculus III; Purple = arcuate fasciculus (MNI, Montreal Neurologic Institute; R, right; L, left; S, superior; I, inferior).

A mask representing the voxels that correspond to the SLF was manually drawn by experienced researchers (SJ and HJ) on anatomically defined areas on the mean FA skeleton map using 3D Slicer (version 4.8.1.). SLF subcomponent masks (SLF I, SLF II, SLF III, and AF) were drawn using the freehand paint tool (intraclass correlation coefficient > 0.95 for all subcomponents). The branches of the SLF were isolated according to well-validated definitions in previous studies^59,60^ (Fig. 4). AF was defined as fibers that did not pass through the anterior–posterior commissure line at the temporal lobe of the axial plane. On the coronal axis, the area passing through the superior frontal gyrus was defined as SLF I, the area passing through the middle frontal gyrus as SLF II, and the area passing through the inferior frontal/precentral gyrus as SLF III.

### Statistical analysis

Independent *t*-tests for continuous variables and chi-squared tests for categorical variables were applied to compare the demographic and clinical characteristics between patients with SZ and HCs. FAs of the SLF were compared between patients with SZ and HCs using voxel-wise comparison analysis in the TBSS. The same comparison analyses were performed for other DTI indices (MDs, ADs, and RDs). A brain mask was generated to specifically include SLF voxels that showed significant FA differences between the patients with SZ and HCs. Voxel-wise correlation analyses were performed for each group to investigate the correlations of FAs in the area in the mask with the FSIQ. Exploratory voxel-wise correlation analyses were additionally performed to investigate the associations of the K-WAIS subtest scores with the FAs of the areas that showed significant results. The same method was applied to TR of NS in patients with SZ. Corresponding SLF subcomponents for areas that showed significant results were then identified by inspections with respect to the ROI mask for the SLF subcomponents.

To further explore the nature of the group differences in WM integrity of manually edited SLF subcomponents between patients with SZ and HCs in addition to voxel-wise analysis, RM-ANOVA was performed (see Supplementary Methods and Results). In addition, to examine the independence of the associations of each K-WAIS subtest score with SLF FAs, multivariate multiple stepwise linear regression analysis was performed. Shapiro–Wilk tests were performed to confirm the normality of the variables. The FAs of the SLF voxels that showed significant correlations with the FSIQ scores were set as dependent variables, and the K-WAIS subtest scores were set as independent variables for the equation. Multivariate multiple stepwise linear regression analysis was performed to investigate the predictive value of FAs in areas that showed a significant association with the TR of NS. The 6-month TR of NS was set as the dependent variable. The FAs of the areas with significant associations as well as age, sex, duration of illness, and baseline PANSS scores were set as explanatory variables. All statistical analyses except for voxel-wise analyses were performed using Statistical Product and Service Solutions, version 26 (IBM Corp., Armonk, NY, USA).

## Supplementary information

Supplementary Methods and Results

Supplementary Figure 1

Supplementary Figure 2

## Data Availability

The data that would be necessary to interpret, replicate and build upon the methods or findings reported in this article are available on request from the corresponding author S.J. The data are not publicly available because of ethical restrictions that protect patients’ privacy and consent.
