# Author Correction: White matter microstructure of superior longitudinal fasciculus II is associated with intelligence and treatment response of negative symptoms in patients with schizophrenia

**DOI:** 10.1038/s41537-022-00266-4

**Published:** 2022-07-07

**Authors:** Joonho Lee, Jong-Soo Oh, Chun-Il Park, Minji Bang, Gihye Sung, Sra Jung, Sang-Hyuk Lee

**Affiliations:** 1 Department of Psychiatry, CHA Bundang Medical Center, CHA University, Seongnam, Republic of Korea; 2 CHA University School of Medicine, Seongnam, Republic of Korea; 3 Department of Psychology, Korea University, Seoul, Republic of Korea; 4 Department of Psychiatry, Kangbuk Samsung Medical Center, Sungkyunkwan University School of Medicine, Seoul, Republic of Korea

**Keywords:** Schizophrenia, Schizophrenia

Correction to: *Schizophrenia* 10.1038/s41537-022-00253-9, published online 27 April 2022

In the version of this article initially published, there were errors in the 2nd paragraph of ‘Clinical assessments’ in the Methods section and in the legend of Supplementary figure 2, where the subtracted values appeared as ‘−30’ rather than ‘−7.’ The errors have been corrected in the HTML and PDF versions of the article.

## Supplementary information

Supplementary figure 2

