# Supplementary material for: White matter microstructure of superior longitudinal fasciculus II is associated with intelligence and treatment response of negative symptoms in patients with schizophrenia
Source: Schizophrenia (Heidelb). 2022 Apr 27;8(1):43. doi: 10.1038/s41537-022-00253-9 (PMC9262917; doi:10.1038/s41537-022-00253-9)
Supplement: Supplementary file 1 — Supplementary Methods and Results [file 41537_2022_253_MOESM1_ESM.docx]

**Supplementary methods and results**

*Methods*

To comprehensively explore the nature of group differences in WM integrity, we compared manually edited SLF subcomponents between patients with SZ and HCs. Additional RM-ANOVA was performed with “group” (patients with SZ vs. HCs) as a between-subjects factor, and “hemisphere” (left vs. right) and “subcomponent” (SLF I, II, III, and AF) as within-subjects factors. *Post-hoc* comparisons for the eight SLF subcomponents were performed.

*Results*

RM-ANOVA revealed that the two groups differed in whole SLF FAs (F1,198 = 19.2, *p* < 0.001) with a significant group-by-hemisphere-by-subcomponent interaction (*F* = 4.34; *p* = 0.005). Follow-up RM-ANOVA showed a significant interaction for group × subcomponent (F3,196 = 8.7, *p* < 0.001), and a subsequent *post hoc* test found significantly lower FAs in all eight SLF subcomponents in patients with SZ (see Supplementary Tables 1 and 2).

A significant interaction for hemisphere × subcomponent was also observed (F3,196 = 890.4, *p* < 0.001). *The post hoc* independent t-test indicated that FAs of the left SLF I were larger than those of the right SLF I (*p* < 0.001), FAs of the left SLF II and left AF were smaller than those in the right hemisphere (*p* < 0.001 for the SLF II and AF), and SLF III had no significant differences between hemispheres (see Supplementary figure 1). No significant group × hemisphere interactions were observed.

| **Supplementary table 1**. Repeated measures ANOVA for “group” (patients with SZ vs. HC), “hemisphere” (left vs. right), and “subcomponents” (SLF I vs. SLF II vs. SLF III vs. AF) interactions in SLF | | | |
| --- | --- | --- | --- |
| ANOVA | F | *p* | n^2^ |
| Group | 19.16 | < 0.001 | 0.088 |
| Hemisphere | 246.51 | < 0.001 | 0.555 |
| Subcomponent | 1396.34 | < 0.001 | 0.955 |
| Hemisphere x Group | 0.21 | 0.648 | 0.001 |
| Subcomponent x Group | 8.70 | < 0.001 | 0.117 |
| Hemisphere x Subcomponent | 890.45 | < 0.001 | 0.932 |
| Hemisphere x Subcomponent x Group | 4.34 | 0.005 | 0.062 |
| ANOVA, analysis of variance; SZ, schizophrenia; HC, healthy control; SLF, superior longitudinal fasciculus; AF, arcuate fasciculus. | | | |

| **Supplementary table 2.** Comparison of FAs between patients with SZ and HCs^a^ | | | | | |
| --- | --- | --- | --- | --- | --- |
| Regions | SZ | HC | Independent t-test | |  |
|  |  |  | *t* | *p* |  |
| Left |  |  |  |  |  |
| SLF I | 0.541 ± 0.023 | 0.552 ± 0.018 | 3.65 | < 0.001 | SZ < HC |
| SLF II | 0.515 ± 0.024 | 0.527 ± 0.023 | 3.50 | 0.001 | SZ < HC |
| SLF III | 0.510 ± 0.025 | 0.520 ± 0.024 | 2.88 | 0.004 | SZ < HC |
| AF | 0.537 ± 0.023 | 0.557 ± 0.023 | 6.35 | < 0.001 | SZ < HC |
| Right |  |  |  |  |  |
| SLF I | 0.531 ± 0.026 | 0.540 ± 0.019 | 2.95 | 0.003 | SZ < HC |
| SLF II | 0.553 ± 0.026 | 0.562 ± 0.020 | 2.70 | 0.007 | SZ < HC |
| SLF III | 0.511 ± 0.025 | 0.524 ± 0.021 | 4.06 | < 0.001 | SZ < HC |
| AF | 0.553 ± 0.025 | 0.571 ± 0.023 | 5.44 | < 0.001 | SZ < HC |
| ^a^ Mean values are presented with standard deviations.  FA, fractional anisotropy; SZ, schizophrenia; HCs, healthy controls; SLF, superior longitudinal fasciculus; AF, arcuate fasciculus. | | | | | |
