# Supplementary material for: White matter microstructure of superior longitudinal fasciculus II is associated with intelligence and treatment response of negative symptoms in patients with schizophrenia
Source: Schizophrenia (Heidelb). 2022 Apr 27;8(1):43. doi: 10.1038/s41537-022-00253-9 (PMC9262917; doi:10.1038/s41537-022-00253-9)
Supplement: Supplementary file 2 — Supplementary Figure 1 [file 41537_2022_253_MOESM2_ESM.pptx]

## Slide 1
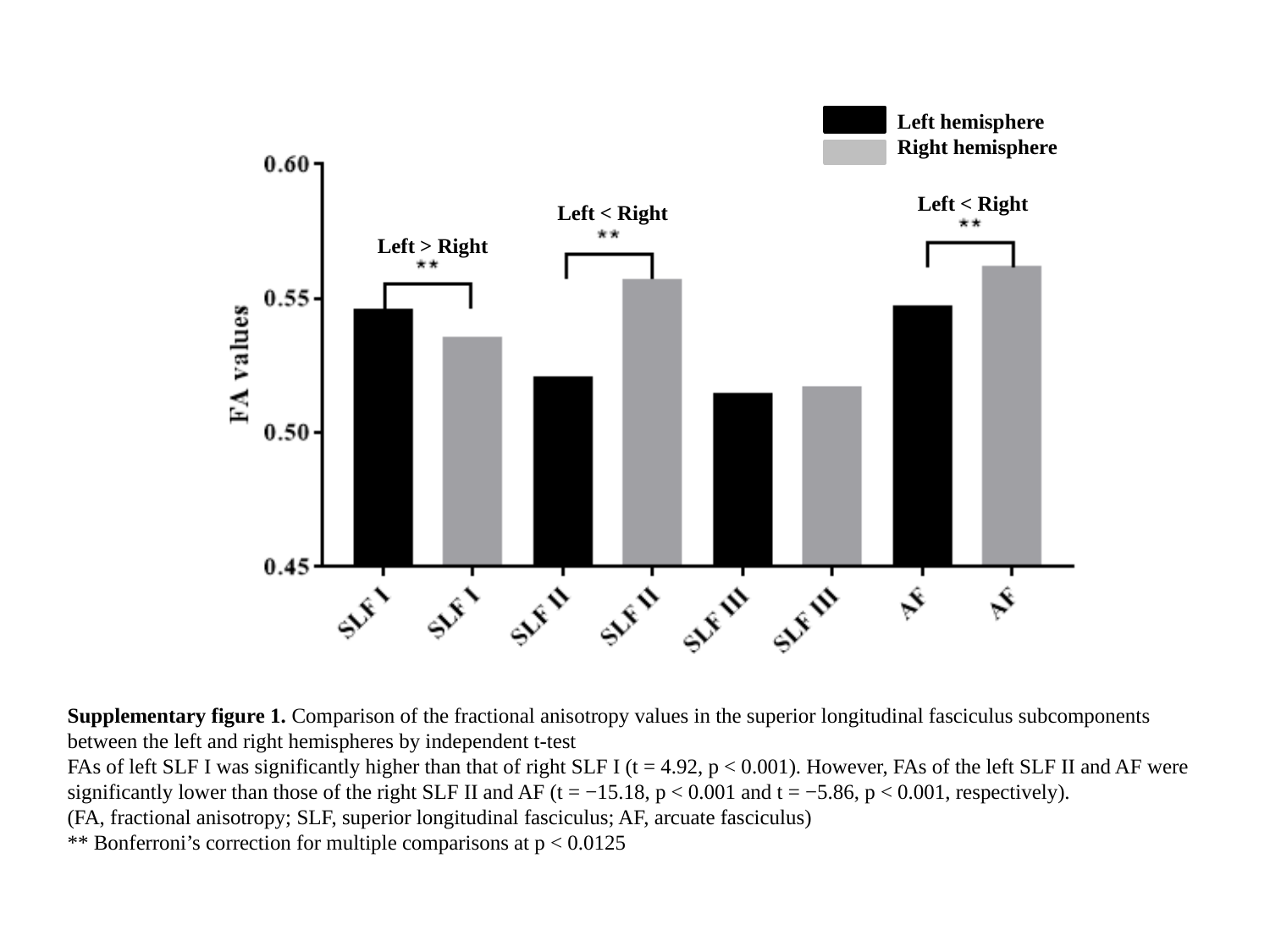

Left hemisphere
Right hemisphere
Left < Right
Left < Right
Left > Right
Supplementary figure 1. Comparison of the fractional anisotropy values in the superior longitudinal fasciculus subcomponents between the left and right hemispheres by independent t-test
FAs of left SLF I was significantly higher than that of right SLF I (t = 4.92, p < 0.001). However, FAs of the left SLF II and AF were significantly lower than those of the right SLF II and AF (t = −15.18, p < 0.001 and t = −5.86, p < 0.001, respectively).
(FA, fractional anisotropy; SLF, superior longitudinal fasciculus; AF, arcuate fasciculus)
** Bonferroni’s correction for multiple comparisons at p < 0.0125
