# Supplementary material for: White matter microstructure of superior longitudinal fasciculus II is associated with intelligence and treatment response of negative symptoms in patients with schizophrenia
Source: Schizophrenia (Heidelb). 2022 Apr 27;8(1):43. doi: 10.1038/s41537-022-00253-9 (PMC9262917; doi:10.1038/s41537-022-00253-9)
Supplement: Supplementary file 3 — Supplementary Figure 2 [file 41537_2022_253_MOESM3_ESM.pptx]

## Slide 1
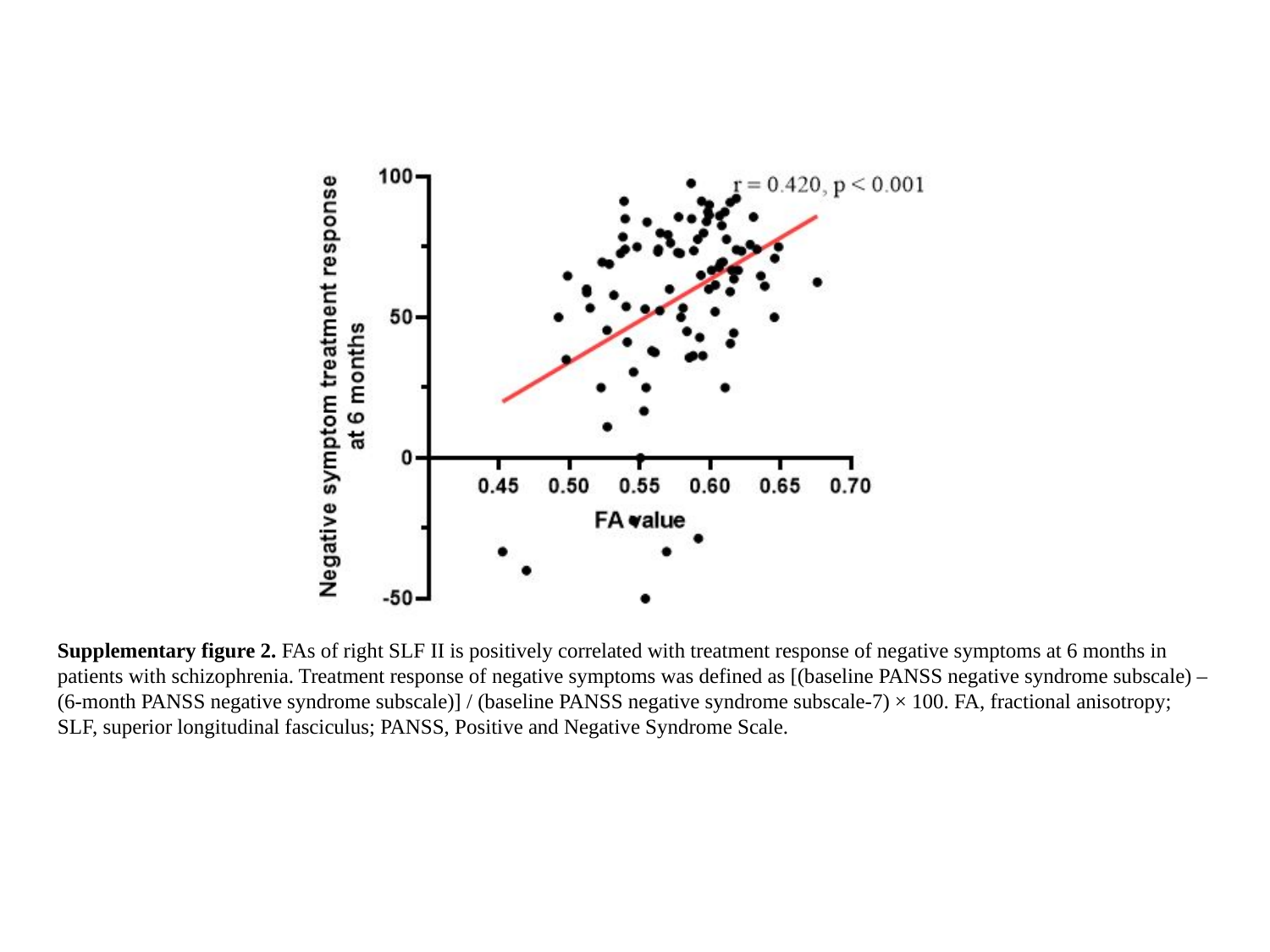

Supplementary figure 2. FAs of right SLF II is positively correlated with treatment response of negative symptoms at 6 months in patients with schizophrenia. Treatment response of negative symptoms was defined as [(baseline PANSS negative syndrome subscale) – (6-month PANSS negative syndrome subscale)] / (baseline PANSS negative syndrome subscale-7) × 100. FA, fractional anisotropy; SLF, superior longitudinal fasciculus; PANSS, Positive and Negative Syndrome Scale.
